# TRP channel activation and cholinergic signaling in insect thermoregulation: evidence from the American cockroach

**DOI:** 10.1007/s00360-026-01699-4

**Published:** 2026-07-27

**Authors:** Justyna Maliszewska, Milena Jankowska, Marianna Artkop, Justyna Rogalska

**Affiliations:** 1 Department of Animal Physiology and Neurobiology, Faculty of Biological and Veterinary Sciences, Nicolaus Copernicus University, Lwowska 1, Toruń, 87-100 Poland; 2 Department of Pathophysiology, Faculty of Pharmacy, Ludwik Rydygier Collegium Medicum, Nicolaus Copernicus University in Toruń, Bydgoszcz, 85-094 Poland

**Keywords:** American cockroach, Capsaicin, MG-624, TRP receptors

## Abstract

Transient receptor potential (TRP) channels mediate insect thermosensation, yet the downstream neurotransmitter pathways linking TRP activation to behavioral output remain unclear. We investigated the role of nicotinic acetylcholine receptors (nAChRs) in thermoregulatory and neural responses induced by TRP ligands in the American cockroach, *Periplaneta americana*. Extracellular recordings from the ventral nerve cord showed that capsaicin reduced spontaneous neuronal firing, whereas the selective α7-containing nAChR antagonist MG 624 increased baseline activity. Notably, nicotinic receptor blockade prevented the capsaicin-induced suppression of neuronal activity, suggesting an interaction between cholinergic signaling and capsaicin-induced neuronal modulation. Behaviorally, capsaicin shifted thermal preference toward cooler temperatures, consistent with TRP-mediated heat avoidance. This response, as well as the effect of the TRPV1 antagonist capsazepine, was markedly attenuated by MG 624 pretreatment. Grooming assays revealed differential modulation of antennal and leg grooming, suggesting distinct neural mechanisms and partial interaction between cholinergic and monoaminergic pathways. Despite pronounced neural and behavioral effects, neither TRP ligands nor nicotinic receptor blockade significantly altered whole-animal metabolic rate, as assessed by CO₂ production. Together, these findings indicate that cholinergic signaling contributes to behavioral responses induced by TRP ligands and interacts with capsaicin-induced neuronal modulation in *P. americana*. The results provide new insight into the neurochemical integration of thermosensory signaling and highlight cholinergic pathways as important contributors to insect thermoregulatory responses.

## Introduction

Transient receptor potential (TRP) channels comprise a diverse superfamily of polymodal cation channels that mediate the detection and integration of environmental stimuli across animal taxa. In insects, TRP channels participate in thermosensation, chemosensation, osmosensation, and mechanosensation, thereby contributing to the maintenance of physiological homeostasis and adaptive behavior. Notably, several insect TRP channels exhibit dual functionality, operating both as thermosensors and chemoreceptors. The TRPA channel Painless, first characterized in *Drosophila melanogaster*, was among the earliest identified insect TRP channels implicated in avoidance of noxious heat (Tracey et al. 2003). Expressed in both central and peripheral components of the nervous system, Painless functions as a high-threshold heat receptor activated by temperatures exceeding 42 °C (Sokabe and Tominaga 2009; Dillon et al. 2009; McKemy 2007). To date, at least twelve TRP family members have been associated with thermosensory processes in insects (Rosenzweig et al. 2008; Kwon et al. 2010; Kohno et al. 2010; Gallio et al. 2011; Turner et al. 2016).

Beyond thermal activation, insect TRP channels respond to a broad spectrum of chemical ligands. Allyl isothiocyanate (AITC), a plant-derived electrophile, activates insect heat-sensitive TRP channels (Mendel et al. 2018). Menthol modulates the activity of TRPM and TRPA channels in *D. melanogaster* (Himmel et al. 2019), while capsaicin has been shown to activate Painless in this species (Li et al. 2020). In addition, our previous studies indicate that capsaicin may act as a functional ligand of TRP thermoreceptors in the American cockroach, *Periplaneta americana* (Maliszewska et al. 2018), suggesting evolutionary conservation of chemosensory–thermosensory integration.

Although numerous studies have described behavioral and physiological responses associated with TRP channel activation in insects, considerably less is known about the neural mechanisms that connect TRP-mediated sensory detection with downstream neurochemical signaling. Our recent findings in *P. americana* demonstrated that pharmacological activation of TRP channels increases octopamine levels (Maliszewska et al. 2024), indicating that TRP-dependent sensory signaling engages broader neuromodulatory networks. Because TRP channels, including Painless, are Ca²⁺-permeable and mediate rapid calcium influx upon activation (Sokabe et al. 2008), stimulation of thermosensory afferents is expected to trigger neurotransmitter release. The increase in octopamine observed following TRP activation suggests that sensory signals originating from TRP-expressing neurons are transmitted through excitatory pathways to downstream octopaminergic circuits. Given that acetylcholine (ACh) is the principal excitatory neurotransmitter in the insect nervous system, nicotinic acetylcholine receptors represents plausible candidates for mediating the transfer of TRP-dependent sensory information to neural networks involved in thermoregulation and behavior.

Therefore, we hypothesize that acetylcholine may participate in the transmission of thermosensory signals generated by TRP channel activation. In *D. melanogaster*, thermal information detected by peripheral sensory neurons is projected to the ventroposterior glomeruli of the antennal lobe, where further processing occurs (Marin et al. 2020). Similar central processing of thermal information has been demonstrated in the cockroach *Periplaneta americana*. Anatomical studies have shown that antennal cold receptor neurons project to specific regions of the deutocerebrum, providing direct sensory input to central thermosensory pathways (Nishikawa et al. 1995), while electrophysiological recordings have identified deutocerebral interneurons that respond to temperature changes detected by the antennae (Nishikawa et al. 1991). Together, these findings demonstrate that thermal cues are processed by dedicated central neural circuits. Evidence from recent molecular and functional studies in *Drosophila* further suggest that cholinergic signaling is involved in this pathway, as thermosensory neuron populations express cholinergic markers and form excitatory connections within central thermosensory circuits (Jin et al. 2021; Li et al. 2022; Corthals et al. 2023; Alpert et al. 2020). Collectively, these observations support the view that acetylcholine contributes to the central processing and integration of thermal information. The insect cholinergic system shares key organizational and molecular features with that of vertebrates, comprising choline acetyltransferase, acetylcholinesterases, high-affinity choline transporters, and two major receptor classes: ionotropic nicotinic acetylcholine receptors (nAChRs) and metabotropic muscarinic acetylcholine receptors (mAChRs) (Grünewald and Siefert 2019).

Insect nAChRs are pentameric ligand-gated ion channels that mediate fast excitatory synaptic transmission and are essential for locomotion, learning, and memory (Thany 2025). Multiple receptor subtypes have been identified in the insect CNS, including pharmacologically distinct α-bungarotoxin-sensitive and α-bungarotoxin-insensitive classes. Homomeric receptors containing α7-like subunits display sensitivity to α-bungarotoxin, analogous to vertebrate α7 nAChRs. In *P. americana*, nAChRs with distinct pharmacological profiles have been described in dorsal unpaired median (DUM) neurons, Df motor neurons, and thoracic ganglia (Thany et al. 2007; Salgado 2021), underscoring the functional heterogeneity of cholinergic transmission in this species.

Despite the well-established role of cholinergic transmission in insect neural function, its contribution to thermoregulation and thermosensory integration remains poorly understood. In the honeybee, *Apis mellifera*, activation of nAChRs has been shown to alter thoracic temperature (Tosi et al. 2016), indicating a functional link between cholinergic signaling and thermal physiology. Furthermore, interactions between ACh and defined neuronal populations, such as those expressing the Allatostatin-C receptor AstC-R2, may coordinate physiological adaptations to thermal stress, including diapause and reproductive dormancy under low-temperature conditions (Meiselman et al. 2022).

The present study investigates whether thermoregulatory behavioral changes induced by TRP ligands, particularly capsaicin, are mediated via cholinergic mechanisms in *P. americana*, with specific emphasis on nicotinic acetylcholine receptors. By elucidating the functional interplay between TRP channels and cholinergic signaling pathways, we aim to clarify the neurochemical mechanisms underlying insect thermosensation and adaptive responses to thermal stress, contributing to a comparative understanding of sensory integration in ectothermic animals.

## Materials and methods

### Insects

Experiments were performed on adult males of the American cockroach, *Periplaneta americana* L. Individuals originated from a laboratory colony maintained at Nicolaus Copernicus University (Toruń, Poland). Insects were housed in plastic containers under controlled environmental conditions (28 ± 2 °C; 12:12 h light: dark photoperiod) with *ad libitum* access to water and standard diet.

### Tested substances

Capsaicin (cat. no. M2028; Merck Life Science, Darmstadt, Germany) was initially dissolved in ethanol and subsequently diluted in physiological saline to a final concentration of 1 µM. The final ethanol concentration in all working solutions was 0.01%; the same vehicle solution served as control. Capsazepine (cat. no. C191; Merck Life Science, Darmstadt, Germany) was prepared analogously. MG 624 (cat. no. 1356; Tocris Bioscience, Bristol, UK), a nicotinic acetylcholine receptor antagonist, was likewise dissolved in ethanol and diluted in saline to 1 µM. Compounds were applied topically in a volume of 10 µL to the dorsal thorax.

### Electrophysiological recordings

Extracellular electrophysiological recordings were made on semi-isolated nerve cord of *Periplaneta americana*. Adult male cockroaches were deprived of head, legs and wings and were mounted ventral side down to the cork stand. Using microscissors, the cuticle, digestive system and accessory gland were removed to exhibit the nerve cord. Preparation was kept wet using physiological saline (in mM: NaCl—210, KCl—3.1, CaCl2—5, MgCl2—5.4, Hepes—5; pH = 7.4 was adjusted with NaOH). Spiker box system (Backyard Brains, An Arbor, MI USA) (Marzullo et al. 2012) was used for recording. Two electrodes were placed in the vicinity of abdominal part of nerve cord and the signal was processed by a differential amplifier. Data was registered using dedicated software SpikeRecorder. The recording was started when the nerve activity remained stable for 3 min., then 5 min. of control pre-treatment recording was performed. The average frequency of pre-treatment recordings was set as 1 for further calculations. In each experiment the tested substance was applied in 20 µL volume and recording was continued for at least next 20 min. Data was then processed in SpikeRecorder software, the frequency of spontaneous extracellular action potential was analyzed, then normalized to pre-treatment recordings.

### Thermal preferences assay

Thermal preference was assessed using a linear temperature gradient apparatus as previously described (Maliszewska et al. 2018). The system consisted of an aluminum trough (60 × 4 × 2 cm). One end was connected to a thermostat (Polyscience 8006A12E) circulating warm water, while the opposite end was connected to a cryostat (Polyscience 9106AA2E) supplying cold water, generating a stable gradient ranging from 10 °C to 40 °C. The trough was divided into 20 equal compartments (3 cm each). Temperatures were verified before each trial using a digital thermometer (UT321, Sinometer Instruments).

Immediately following topical treatment, insects were placed in the central compartment (~ 24 °C). For combined treatments, MG 624 was administered 30 min prior to capsaicin or capsazepine application. Each assay involved a single insect per gradient apparatus. Individuals remained in the gradient for 24 h. Behavioral activity was recorded using digital video cameras (SONY HDR-XR200VE or SONY FDR-AX33) and BioVid software (FERRO Software). Twenty-second video segments were captured every 160 s. Insect position within the gradient was subsequently analyzed to determine preferred temperature over time.

### Grooming behavior

Grooming was quantified during a 30-min observation period preceded by a 5-min habituation phase. Individual cockroaches were transferred to glass observation chambers immediately after topical treatment with capsaicin (1 µM, 10 µL), MG-624 (1 µM, 10 µL), or vehicle (0.01% ethanol in saline, 10 µL). In pretreatment experiments, MG 624 was applied 15 min before capsaicin. Behavioral assays were conducted between 2 and 8 h after the onset of the light phase. Ambient temperature during testing was maintained at 23–25 °C. Time spent grooming the antennae and legs was measured manually using a stopwatch during the 30-min observation period.

### Metabolic rate

Metabolic rate was estimated by measuring carbon dioxide production (V̇CO₂) using an infrared CO₂ analyzer (S157, Qubit Systems Inc., Kingston, Canada) calibrated with certified reference gas (500 ppm CO₂). The analyzer operated within a flow-through respirometry system. Compressed ambient air (21% O₂, 79% N₂) was drawn through 5 mL glass respirometry chambers using a pump (P652, Qubit Systems Inc.) at a constant flow rate of 150 mL min⁻¹. Water vapor was removed from the air stream using magnesium perchlorate.

Prior to measurements, insects were deprived of food for approximately 2 h to minimize postprandial metabolic effects. Each individual was weighed (Radwag WTB 200 analytical balance) immediately before testing. Following treatment, cockroaches were placed individually into chambers for a 2-h recording session. Baseline measurements (empty chamber) were taken for 3 min every 15 min to correct for system drift. All measurements were conducted at 25 ± 2 °C.

### Data analysis

Electrophysiological data were analyzed using the Kruskal–Wallis test followed by Mann–Whitney U tests. Thermal preference data were analyzed using a two-way General Linear Model (GLM), with treatment as a fixed factor and time (24 h divided into 2-h intervals) as a repeated-measures factor. Post hoc comparisons were conducted using the Least Significant Difference (LSD) test. Grooming data were log-transformed to meet assumptions of normality and analyzed by one-way ANOVA followed by LSD post hoc tests. Carbon dioxide production was analyzed using ANCOVA, with body mass included as a covariate. All statistical analyses were performed using IBM SPSS Statistics 25 (IBM Corp., Armonk, NY, USA). Differences were considered statistically significant at *p* < 0.05.

## Results

### Nicotinic receptor blockade attenuates the electrophysiological response to capsaicin

**Fig. 1:**
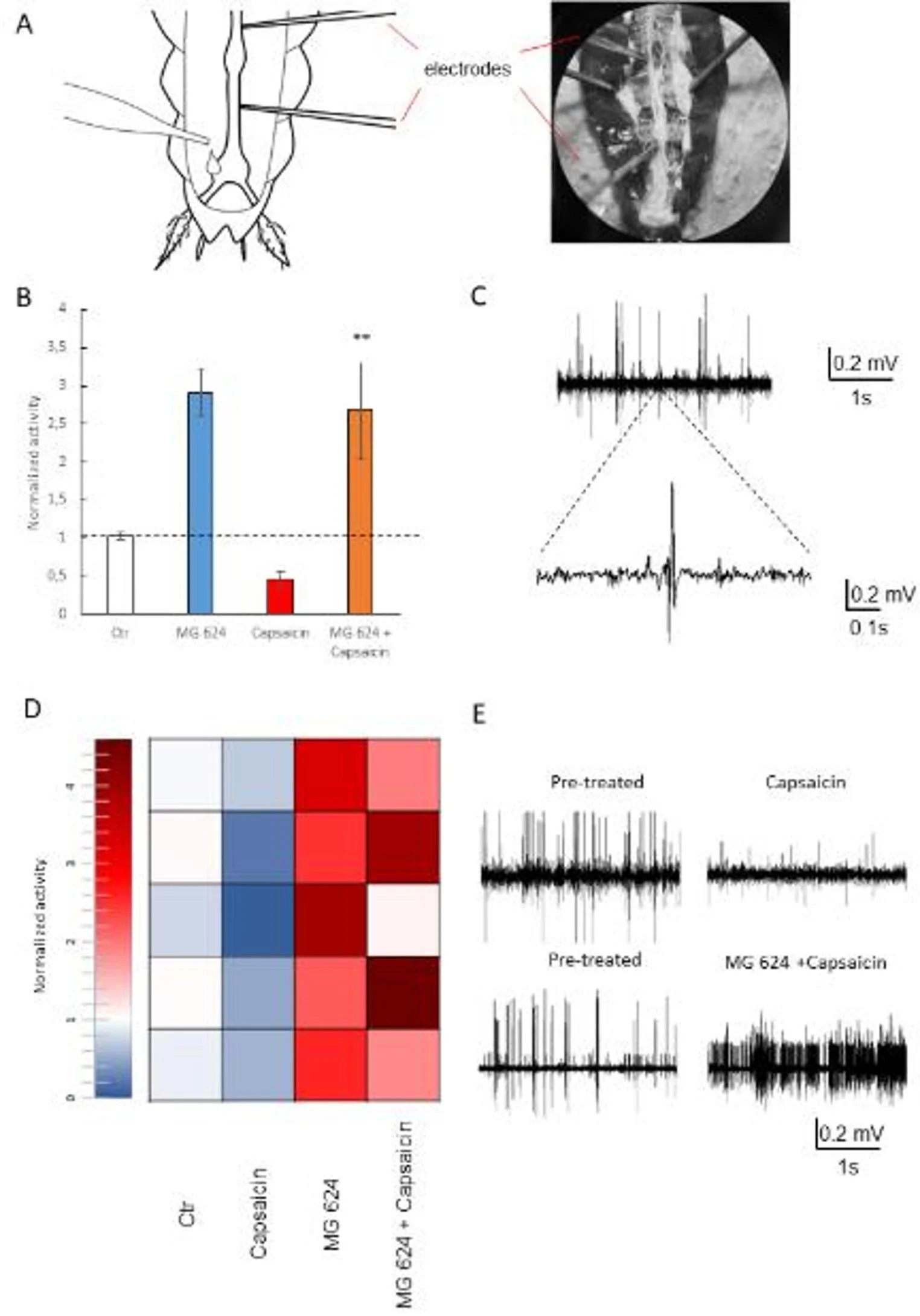
Spontaneous extracellular activity of American cockroaches nerve cord in the presence of capsaicin (1µM), and nicotinic receptor blocker (MG 624) (1µM). **A** Schematic representation of *Periplaneta americana* preparation and corresponding photograph. In-situ preparation consist of peripheral sensory cercal neurons, last abdominal ganglion and abdominal connectives leaving the ganglion. Placement of recording electrodes has been indicated. **B** Normalized activity of nerve cord, as frequency of spontaneous extracellular action potentials divided by pre-treated nerve activity from 5 min of recordings before application of chemicals. Data presented for control (Ctr; *N* = 19) capsaicin (1µM; *N* = 7), nicotinic receptor blocker (MG 624; *N* = 5), and pre-treated with MG 624 before capsaicin (*N* = 5), was collected 13 min after application, when all the chemicals exerted the full effect, and is presented as mean change ± sem. **Indicates statistically significant difference to cockroaches exposed to capsaicin only, *p* = 0.0058. **C** The representative recording of spontaneous extracellular action potentials at different time bases. **D** The representation of nerve activity of each recording (*N* = 5) expressed as a color gradient where blue indicates a decrease in nerve activity and red shows an increase in nerve activity. **E** The representative examples of original recordings of pre-treated nerve activity and 13 min after capsaicin (1µM), administration or capsaicin (1µM) administration with pre-incubation with MG 624

Direct application of capsaicin produced a marked suppression of spontaneous extracellular activity in the abdominal nerve cord of *Periplaneta americana*. Mean spike frequency decreased by approximately 45% relative to baseline values (*p* < 0.001; Fig. 1).

In contrast, treatment with the nicotinic acetylcholine receptor antagonist MG 624 alone significantly increased spontaneous neuronal firing. During MG 624 exposure, spike frequency reached 2.91-fold of control levels, indicating a pronounced elevation of baseline excitability (Fig. 1). These results suggest that nicotinic acetylcholine receptors contribute to the regulation of resting neuronal activity within the ventral nerve cord and that their blockade alters the balance of neuronal excitability in this network. When MG 624 was administered prior to capsaicin, the typical capsaicin-induced suppression of neuronal activity was abolished (*p* = 0.01). Under these conditions, spike frequency remained comparable to that observed following MG 624 alone, suggesting an interaction between the effects of MG 624 and capsaicin on spontaneous neuronal activity.

### Behavioral responses to TRP ligands depend on intact nicotinic signaling

**Fig. 2:**
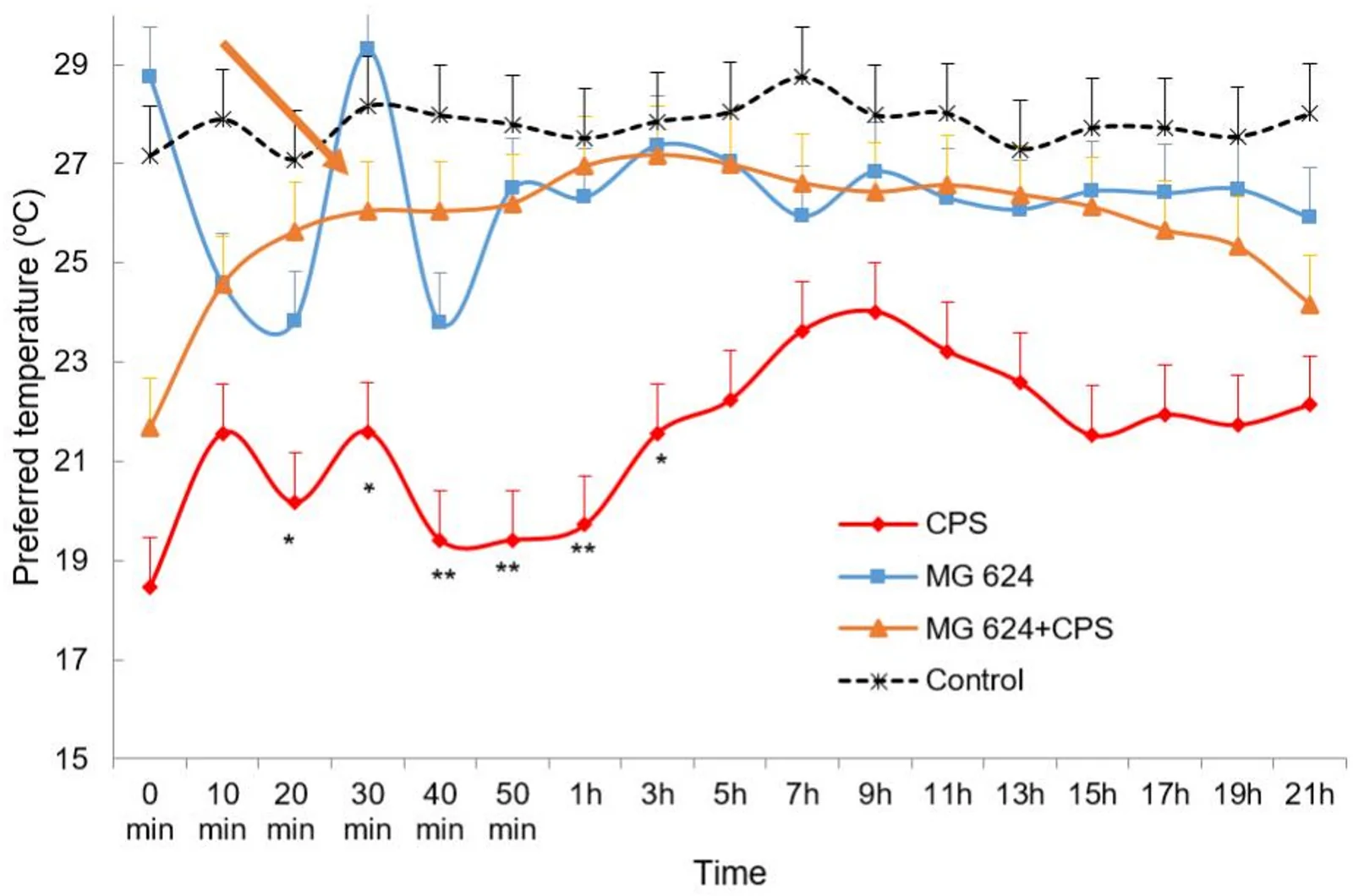
Temperature preferred (° C ± s.e.m) by American cockroaches in control group (Control; *N* = 12) and treated with capsaicin (CPS; 1µM; *N* = 6), MG 624 (1µM; *N* = 5) and pre-treated with MG 624 before capsaicin application (MG 624 + CPS; *N* = 12). Arrow shows time of capsaicin application to cockroaches pre-treated with MG 624. *Indicates significant difference between CPS and MG 624 + CPS (GLM with repeated measures: *p < 0.05; **p < 0.01)

Consistent with electrophysiological findings, behavioral thermoregulation was significantly affected by pharmacological manipulations (GLM: *F* = 2.76; df = 2; *p* = 0.04). Capsaicin treatment induced a shift in thermal preference toward cooler regions of the gradient (Fig. 2). However, this effect was completely abolished by pretreatment with MG 624. In fact, insects receiving MG 624 prior to capsaicin selected significantly higher temperatures during the first 3 h of observation compared to the capsaicin-only group.

**Fig. 3:**
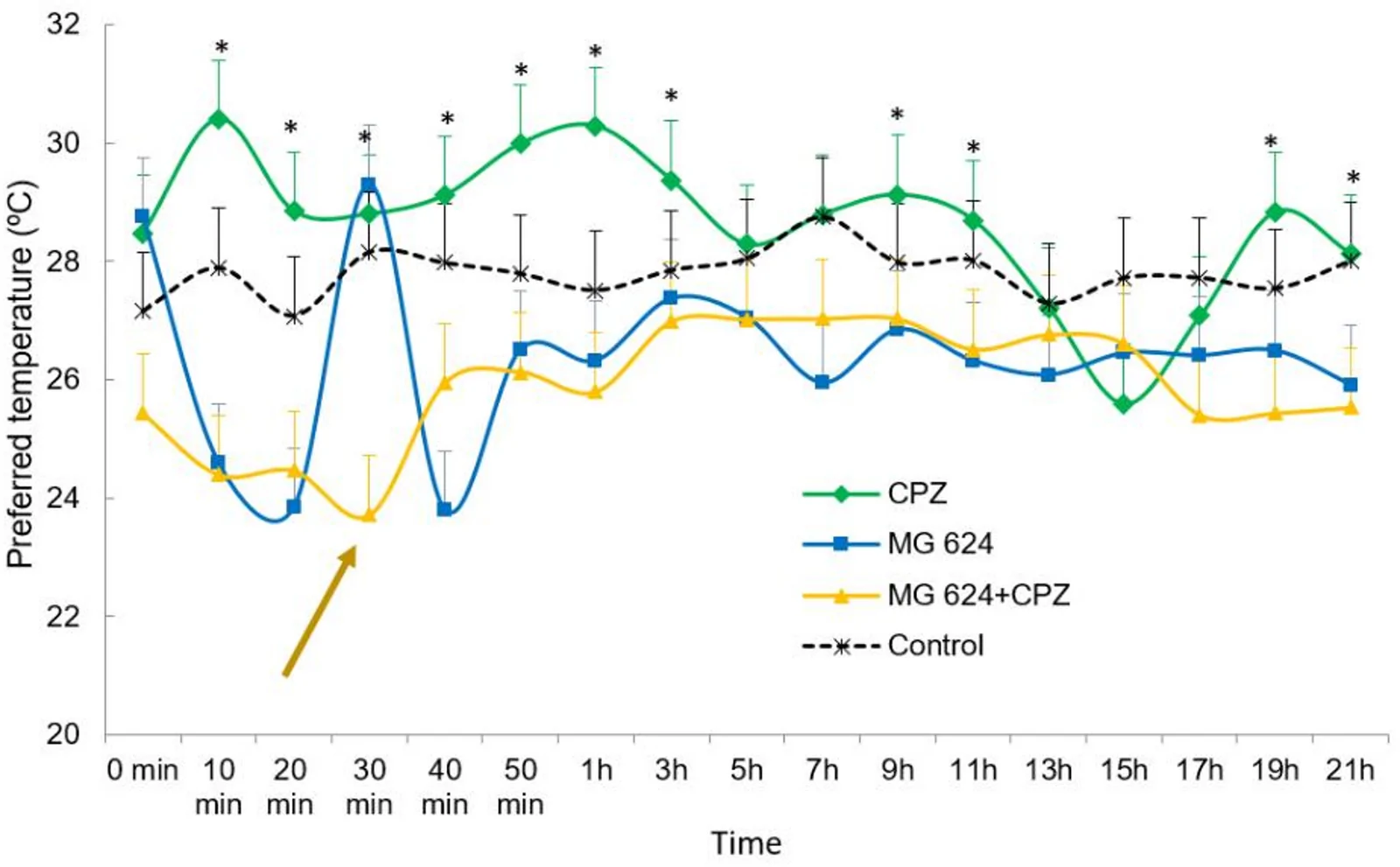
Temperature preferred (° C ± s.e.m) by American cockroaches in control group (Control; *N* = 12) and treated with capsazepine (CPZ; 1µM; *N* = 6), MG 624 (1µM; *N* = 5) and pre-treated with MG 624 before capsaicin application (MG 624 + CPZ; *N* = 6). Arrow shows time of capsazepine application to cockroaches pre-treated with MG 624. *Indicates significant difference between CPZ and MG624 + CPZ (GLM with repeated measures: *p < 0.05; **p < 0.01)

To determine whether this interaction was specific to capsaicin-induced TRP activation, we examined the effects of capsazepine (CPZ), a TRPV1 antagonist. Capsazepine alone induced preference for higher ambient temperatures. However, combined administration of MG 624 and capsazepine significantly attenuated this effect (GLM: *F* = 8.3; df = 2; *p* = 0.004). Cockroaches treated with both compounds selected cooler temperatures than those exposed to capsazepine alone (Fig. 3), indicating that nicotinic signaling contributes broadly to TRP-dependent thermoregulatory behavior. In the combined-treatment groups (MG 624 + CPS and MG 624 + CPZ), the values recorded at 0 min were obtained after MG 624 administration but before the application of capsaicin or capsazepine. Therefore, any differences observed at this time point cannot be attributed to the TRP ligands and most likely reflect natural inter-individual variability among the independently assigned groups.

### Grooming behavior

**Fig. 4:**
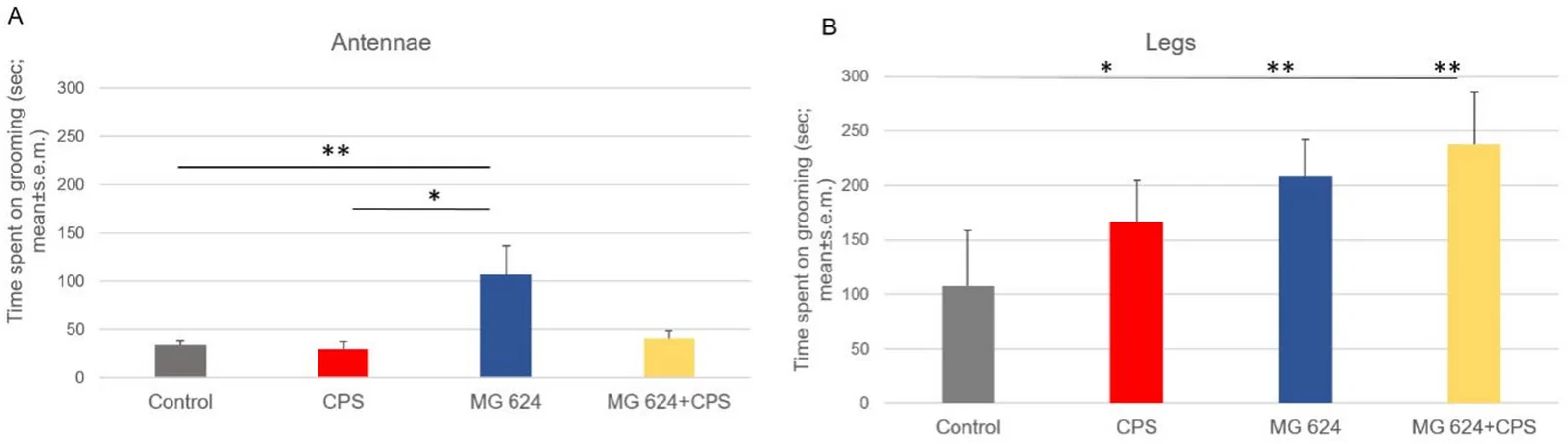
Time (sec) (mean ± s.e.m.) spent on antennae (**A**) or leg (**B**) grooming in cockroaches treated with capsaicin (CPS; 1µM; *N* = 8), MG 624 (1µM; *N* = 8) and pre-treated with MG 624 before capsaicin application (MG 624 + CPS; *N* = 8). * indicates significant difference between groups (one-way ANOVA, post hoc LSD test: * *p* < 0.05; ** *p* < 0.01)

Antennal grooming was not significantly altered by capsaicin alone (29.8 ± 7.8 s; 86% of control levels). In contrast, MG 624 markedly increased antennal grooming (106.6 ± 30.3 s), representing approximately a threefold increase over control values and 3.59-fold over the capsaicin group (*p* = 0.02; Fig. 4A).

Pretreatment with MG 624 prior to capsaicin did not significantly differ from capsaicin alone (*p* = 0.051), suggesting that nicotinic receptor blockade does not further enhance capsaicin-induced antennal grooming under these conditions.

Leg grooming was significantly elevated following both capsaicin (*p* = 0.04) and MG 624 (*p* = 0.009) administration compared to controls. Cockroaches pretreated with MG 624 and subsequently exposed to capsaicin spent 237.7 ± 48 s grooming their legs, corresponding to a 2.2-fold increase relative to controls (*p* = 0.006). However, no significant differences were detected between capsaicin alone, MG 624 alone, and the combined treatment (Fig. 4B).

### Metabolic rate is not significantly affected by TRP or nicotinic stimulation

Given that both thermoregulatory shifts and grooming involve sustained motor activity, we examined whether these behavioral and electrophysiological effects were accompanied by changes in metabolic rate, as estimated by CO₂ production.

**Fig. 5:**
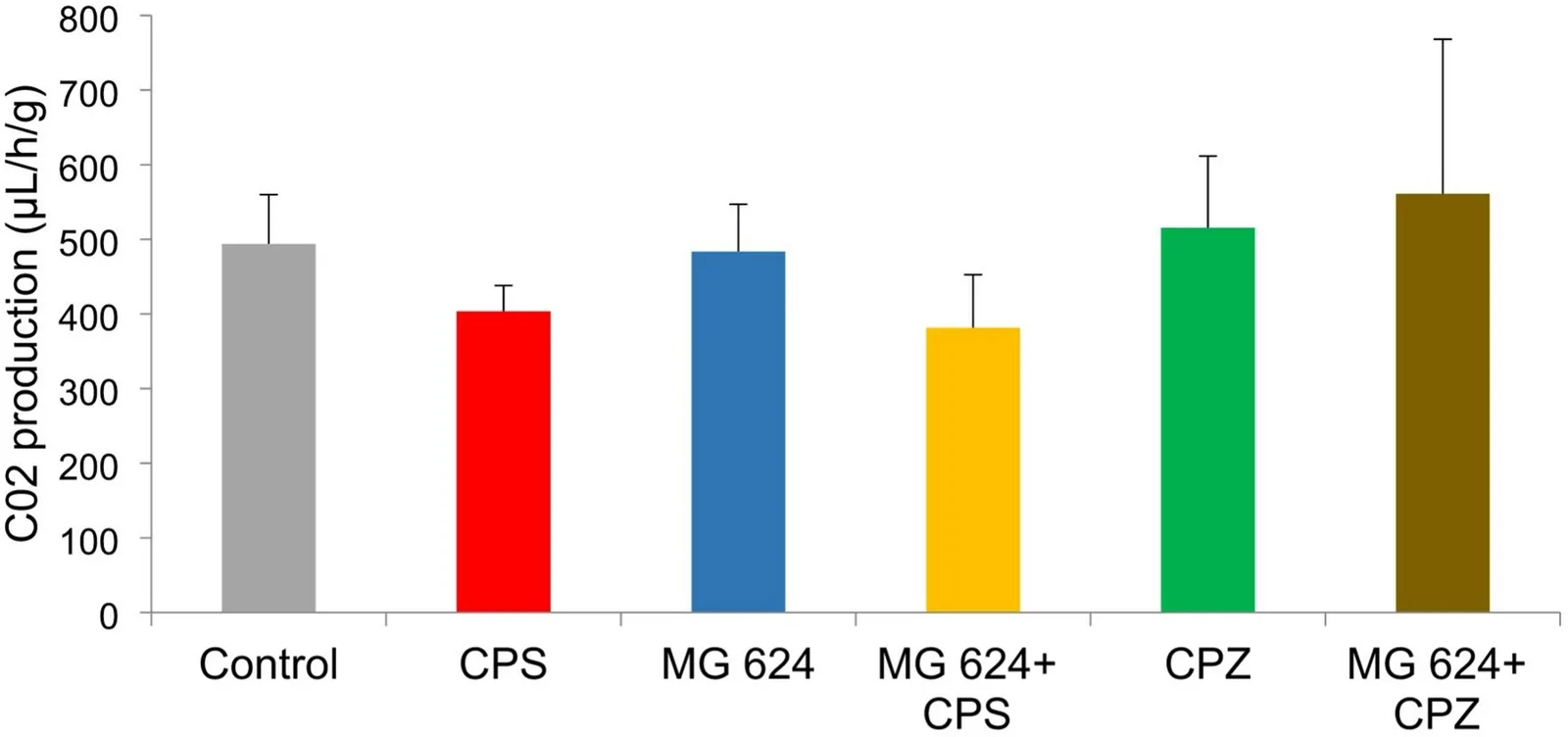
C0_2_ production (µL h⁻¹ g⁻¹ ±s.e.m.) in American cockroaches treated with capsaicin (CPS; 1µM; *N* = 15), MG 624 (1µM; *N* = 15), capsazepine (CPZ; 1µM; *N* = 12), and pre-treated with MG 624 before capsaicin application (MG 624 + CPS; *N* = 12) or capsazepine application (MG 624 + CPZ; *N* = 12)

No statistically significant differences in mass-specific CO₂ production were observed among treatment groups. Mean CO₂ production in controls was 493.86 ± 66.11 µL h⁻¹ g⁻¹. Treated groups ranged from 381.56 ± 71.1 µL h⁻¹ g⁻¹ (MG 624 + CPS) to 560.95 ± 207.17 µL h⁻¹ g⁻¹ (MG 624 + CPZ). Although capsaicin alone (403.67 ± 34.33 µL h⁻¹ g⁻¹) and MG 624 + capsaicin (381.56 ± 71.1 µL h⁻¹ g⁻¹) showed numerically lower values than controls, and capsazepine-treated groups exhibited higher means, none of these differences reached statistical significance. Thus, neither TRP modulation nor nicotinic receptor blockade produced consistent or robust alterations in whole-animal metabolic rate under the present experimental conditions (Fig. 5).

## Discussion

Thermosensation in insects relies on a distributed network of molecular sensors and neural circuits that integrate peripheral thermal input with central processing. Although the involvement of TRP channels in insect thermodetection is well established, considerably less is known about the downstream neurotransmitter systems that translate TRP activation into coordinated physiological and behavioral responses. In the present study, we investigated the contribution of the cholinergic system—specifically nicotinic acetylcholine receptors (nAChRs)—to behavioral and electrophysiological effects induced by TRP ligands in the American cockroach, *Periplaneta americana*. Our findings provide evidence that nicotinic signaling constitutes an important component of TRP-dependent thermoregulatory responses.

To probe the involvement of nAChRs, we employed MG 624, a selective antagonist of neuronal α7-subunit–containing receptors (Gotti et al. 1998). Knowledge regarding the pharmacological properties of α7-like receptors in insects remains limited. Previous unpublished work from our laboratory demonstrated that MG 624 significantly reduced stimulus-evoked compound action potentials recorded from the connective exiting the terminal abdominal ganglion. In the cockroach escape circuit, such stimulus-evoked responses are brief and precisely timed, mediating rapid transmission of sensory information to trigger escape behavior. Comparable roles of α7-containing nAChRs have been described in other insects. In *Drosophila melanogaster*, α7 subunits contribute to visually mediated escape circuits (Fayyazuddin et al. 2006). A defining characteristic of α7 receptors is their rapid desensitization upon acetylcholine binding. This kinetic profile is consistent with our earlier observations that stimulus-evoked responses are short-lived and abruptly terminate, likely reflecting strong presynaptic acetylcholine release followed by rapid receptor inactivation. Such dynamics enable the generation of neural signals clearly distinguishable from spontaneous background activity. In the present experiments, administration of MG 624 induced a rapid increase in the frequency of spontaneous activity recorded from the ventral nerve cord. At first glance, this finding may appear unexpected, given that acetylcholine serves as the main excitatory neurotransmitter in the insect nervous system and antagonism of nAChRs would generally be predicted to reduce neural activity. However, the signals recorded from the abdominal connectives reflects the integrated output of a complex neural network that includes sensory afferents, giant interneurons, and dorsal unpaired median (DUM) neurons, whose intrinsic pacemaker activity is strongly modulated by multiple neurotransmitter systems (Pollack et al. 1988; Dubreil et al. 1994; Geffard et al. 1996). nAChR receptors are not only present in synapses between sensory neurons and giant interneurons, but also they are widely expressed in DUM neurons, which influence spontaneous activity of the escape system of the cockroach.

Although MG 624 has not been extensively characterized in insects, its pharmacological activity is known to be associated with nicotinic acetylcholine receptors. Therefore, the increase in spontaneous activity observed in the present study is more likely to reflect the complexity of cholinergic regulation within the cockroach neural network than a non-specific action of the compound. In particular, the documented involvement of nicotinic receptors in the modulation of DUM neuron excitability (Grolleau et al. 1996) provides a plausible mechanism through which nicotinic receptor blockade may alter the overall spontaneous activity recorded from the abdominal connectives. Therefore, the increase in spontaneous firing observed after MG 624 administration may reflect network-level effects resulting from modulation of cholinergic signaling within the escape circuitry rather than a simple reduction of excitatory transmission. The subsequent return toward control levels suggests the involvement of compensatory mechanisms or additional receptor populations contributing to the regulation of spontaneous activity.

Capsaicin, in contrast, significantly reduced spontaneous neural activity, consistent with recent findings that capsaicin can inhibit slow-inactivating sodium currents in *P. americana* neurons (Kamezaki and Nishiwaki 2025).

When capsaicin was applied in the presence of MG 624, the reduction in spontaneous firing was no longer observed. We acknowledge that, because MG 624 alone exerted a pronounced effect on spontaneous activity, the present data do not allow us to determine whether nicotinic receptor signaling is required for the electrophysiological action of capsaicin. The absence of a capsaicin-induced decrease in activity following MG 624 treatment may reflect an interaction between cholinergic signaling and the mechanisms engaged by capsaicin. However, an alternative explanation is that the strong effect of MG 624 on network activity obscured the suppressive action of capsaicin. Therefore, our data support an interaction between MG 624-sensitive pathways and capsaicin-induced neuronal modulation, but they do not establish the precise nature of this interaction.

Behavioral data are consistent with, and complement the electrophysiological observations, suggesting that the interactions between TRP-dependent signaling and cholinergic pathways may contribute to thermoregulatory behavior. Consistent with our previous report (Maliszewska et al. 2018), capsaicin induced a shift toward cooler ambient temperatures and reduces head temperature, paralleling hypothermic and heat-avoidance responses observed in mammals following TRPV1 activation (Szolcsányi 2015). In cockroaches, capsaicin promotes selection of cooler environments. Importantly, these thermoregulatory effects were abolished when nicotinic receptors were blocked with MG 624. Moreover, the behavioral response to capsazepine, a TRPV1 antagonist, was likewise attenuated by nicotinic blockade. Together, these findings suggest that cholinergic signaling contributes to the behavioral consequences of TRP receptor modulation. It should be noted that, in the groups receiving combined treatments, capsaicin or capsazepine was applied after the start of the behavioral assay. While vehicle controls indicate that the application procedure alone was not sufficient to induce the observed changes in thermopreference, a minor contribution of handling or application-related effects cannot be entirely excluded and should be considered when interpreting these results.

A further point to consider is that the electrophysiological and behavioral experiments differed in the route of drug administration and level of biological organization. In the electrophysiological experiments, compounds were applied to a semi-isolated preparation in which the ventral nerve cord was exposed while peripheral sensory structures remained intact, allowing access to both central and peripheral neural elements. In contrast, capsaicin, capsazepine, and MG 624 were applied externally in the behavioral assays. Although numerous biologically active compounds are known to penetrate the insect cuticle and affect internal target tissues (Tak and Isman 2015; Yahouédo et al. 2027), the present experiments do not allow us to determine where capsaicin, capsazepine, or MG 624 exerted their primary effect. Consequently, it remains unclear whether the observed behavioral responses resulted from action on peripheral sensory structures, central neural circuits, or a combination of both. The results clearly show that interference with nicotinic signaling alters the thermoregulatory effects of TRP ligands, however the exact anatomical site at which these interactions occur remains to be established.

Grooming behavior provided additional insight into the neurochemical pathways involved. In insects, grooming is strongly influenced by neuromodulators. For example, monoaminergic manipulation can profoundly alter grooming patterns; reserpine-induced monoamine depletion elicits intense grooming in cockroaches (Liebersat and Pflueger 2004), and octopamine enhances leg grooming (Carrazoni et al. 2016). We previously demonstrated that capsaicin elevates octopamine levels and increases leg grooming in *P. americana* (Maliszewska et al. 2024). In the present study, capsaicin produced only minor changes in antennal grooming but markedly increased leg grooming. MG 624 alone strongly enhanced antennal grooming, whereas combined MG 624 and capsaicin treatment did not further augment this parameter. In contrast, leg grooming progressively increased across treatments, with the highest values observed under combined exposure. These divergent patterns suggest that antennal and leg grooming are governed by partially distinct neural circuits and receptor populations. Although dopamine and octopamine are well established modulators of grooming behavior (Novikova and Zhukovskaya 2015; Borbor et al. 2025), comparatively little is known about the role of acetylcholine. Studies in honeybees have shown that acetylcholinesterase inhibitors increase grooming activity (Williamson et al. 2013), indicating that cholinergic signaling may influence motor stereotypies. Because MG 624 selectively targets α7-containing receptors without affecting other subtypes such as α4β2- or α3β4-like receptors, differential receptor distribution may account for the distinct grooming responses observed between antennae and legs. It is also plausible that capsaicin-induced octopaminergic modulation exerts a stronger influence on leg grooming, thereby overshadowing cholinergic effects in this behavioral domain.

Despite pronounced electrophysiological and behavioral alterations, neither capsaicin, capsazepine, nor MG 624 significantly affected whole-animal metabolic rate. This finding indicates that modulation of neural excitability and thermoregulatory behavior does not necessarily translate into measurable changes in global energy expenditure under the present experimental conditions. The absence of metabolic effects suggests that the observed responses primarily reflect neural circuit reconfiguration rather than systemic metabolic adjustments.

Collectively, our results indicate that cholinergic signaling contributes to behavioral responses induced by TRP ligands in *P. americana* and that both capsaicin and nicotinic receptor blockade can modulate neuronal activity within the cockroach nervous system. While the electrophysiological and behavioral experiments address different levels of biological organization and do not establish a direct mechanistic link between peripheral thermosensory signaling and central neuronal modulation, they collectively highlight interactions between TRP ligand-dependent responses and cholinergic pathways. The present findings suggest that nicotinic acetylcholine receptors influence the behavioral effects of capsaicin and capsazepine, although the precise site and mechanism of this interaction remain to be determined. Further studies will be required to establish how TRP-dependent sensory signals are integrated with cholinergic neurotransmission in the neural circuits underlying thermoregulatory behavior in insects.

## Data Availability

The data that support the findings of this study are available from the corresponding author upon request.
